# Ultrasonographic assessment of the muscle mass of the rectus femoris in mechanically ventilated patients at intensive care unit discharge is associated with deterioration of functional status at hospital discharge: a prospective cohort study

**DOI:** 10.62675/2965-2774.20250050

**Published:** 2025-01-30

**Authors:** Thiele Cabral Coelho Quadros, Thaline Lima Horn, Marina Santos de Moraes, Luisa da Cunha Selmo, Alexandre Ribas, Clarissa Netto Blattner, Márcio Manozzo Boniatti

**Affiliations:** 1 Pontifícia Universidade Católica do Rio Grande do Sul Hospital São Lucas Physiotherapy Department Porto Alegre RS Brazil Physiotherapy Department, Hospital São Lucas, Pontifícia Universidade Católica do Rio Grande do Sul - Porto Alegre (RS), Brazil.; 2 Universidade Federal do Rio Grande do Sul Hospital das Clínicas de Porto Alegre Porto Alegre RS Brazil Hospital das Clínicas de Porto Alegre, Universidade Federal do Rio Grande do Sul - Porto Alegre (RS), Brazil.

**Keywords:** Critical illness, Muscle weakness, Quadriceps muscle, Ultrasonography, Functional status, Physical functional performance, Patient discharge, Respiration, artificial, Intensive care units

## Abstract

**Objective:**

To verify whether the rectus femoris muscle mass in mechanically ventilated patients assessed by ultrasonography at intensive care unit discharge is associated with functional status at hospital discharge.

**Methods:**

This cohort study was conducted at a tertiary hospital in Brazil between August 2019 and November 2020. We included patients over 18 years who were previously independent (Barthel index > 60) and underwent mechanical ventilation for at least 48 hours within 96 hours of admission. Ultrasonographic measurements of the rectus femoris cross-sectional area and right quadriceps thickness were performed upon enrollment, five days after enrollment, and at intensive care unit discharge. The primary outcome was assessing functional capacity via the Barthel index at hospital discharge.

**Results:**

Of the 78 patients included, 35 had assessable primary outcomes. Twenty (57.1%) patients were considered functionally dependent (Barthel index < 60). The Barthel index at hospital discharge was correlated with the cross-sectional area (r = 0.53; p = 0.001) and quadriceps thickness (r = 0.43; p = 0.01) at intensive care unit discharge. Multiple linear regression analysis revealed that the cross-sectional area at intensive care unit discharge was independently associated with the Barthel index.

**Conclusion:**

We found that muscle mass assessed by cross-sectional area ultrasonography at intensive care unit discharge was significantly correlated with functional capacity at hospital discharge.

## INTRODUCTION

Up to two-thirds of critically ill patients are diagnosed with intensive care unit-acquired weakness (ICU-AW), which is characterized by generalized, bilateral weakness that affects both the peripheral and respiratory muscles.^(1)^ Intensive care unit-acquired weakness leads to negative outcomes, including prolonged mechanical ventilation (MV), longer hospital stays, decreased muscle strength, impaired functional capacity, dependence on activities of daily living (ADLs), and reduced quality of life.^(2)^ The risk factors for ICU-AW development are associated with patient characteristics, such as age, comorbidities, and disease severity, as well as instituted treatment, such as administering neuromuscular blockers and corticosteroids.^(3)^ These factors result in prolonged bed rest and inadequate nutritional support,^(1,4,5)^ which contributes to the development of ICU-AW.

Some studies have demonstrated that physical therapy intervention facilitates the prevention and rehabilitation of critically ill patients, increases muscle strength at intensive care unit (ICU) discharge, and maintains functionality after hospital discharge.^(6,7)^ However, recent randomized controlled trials (RCTs) of physical rehabilitation in the ICU have not shown any appreciable short- or long-term functional improvements.^(8,9)^ Subject heterogeneity is one theory that accounts for these findings. Thus, identifying patients at increased risk of functional disability is important for the appropriate allocation of rehabilitation interventions.^(10)^ As a tool to evaluate and monitor changes in skeletal muscle, muscle ultrasonography (US) has received attention as a low-cost, noninvasive test with adequate reproducibility to help better classify patients at risk for physical limitations.^(11-13)^ Mayer et al. demonstrated that muscle US parameters measured in the ICU are significant predictors of physical function at hospital discharge.^(14)^ More recently, in a cohort of COVID-19 ICU patients, a good correlation was found between muscle US and validated muscle mass parameters, suggesting that muscle US could be an important tool for rapid bedside prognostic evaluation of critical patients after discharge from the ICU.^(15)^

The aim of this study was to verify whether the rectus femoris muscle mass in mechanically ventilated patients assessed by US at ICU discharge is associated with functional status at hospital discharge. Additionally, we aimed to determine whether there was any loss of muscle mass in the first five days of hospitalization and to investigate its potential association with the outcome.

## METHODS

This single-center cohort study was conducted between August 2019 and November 2020 in the medical-surgical ICU of a tertiary hospital in Porto Alegre, Brazil. The ICU has 59 beds. The study was approved by the Ethics Committee of the *Hospital Sao Lucas* da *Pontifícia Universidade Católica do Rio Grande do Sul* (CAAE 13150419200005336), and all legal representatives signed a free consent form.

The study included a convenience sample of patients older than 18 years who were previously functional (Barthel Index [BI] > 60), had been admitted to the ICU, had undergone MV for at least 48 hours, and were enrolled within 96 hours of admission. Patients with neuromuscular diseases, amputations, or those who died before hospital discharge were excluded. The institution's nutritional support is in line with the ASPEN guidelines.^(16)^ All patients underwent a daily sedation review and reduction when appropriate. Physiotherapists perform respiratory and motor physiotherapy as routine care two to three times a day throughout the entire ICU stay. In the ward, physiotherapy care was provided once or twice a day.

Upon inclusion in the study, the following variables were collected: age, sex, reason for admission, Simplified Acute Physiology Score 3 (SAPS 3), Sequential Organ Failure Assessment (SOFA) score, and BI before ICU admission. The BI, relating to the preadmission period, was completed by a family member. Throughout the ICU stay, data on MV duration, length of stay, vasopressor usage, corticosteroid administration, use of neuromuscular blockers, and the need for renal replacement therapy were documented. Upon ICU discharge, orthostatic capacity was assessed and categorized as passive, active-assisted, or active. Patient follow-up continued until hospital discharge.

Upon awakening from sedation, compliant patients capable of responding to at least three of the five De Jonghe commands^(17)^ underwent the Medical Research Council (MRC) test. This test was used to assess six muscle groups, ranging from 0 to 5, with a maximum total score of 60 points. A score below 48 was suggestive of ICU-AW.^(18)^

**Figure 1:**
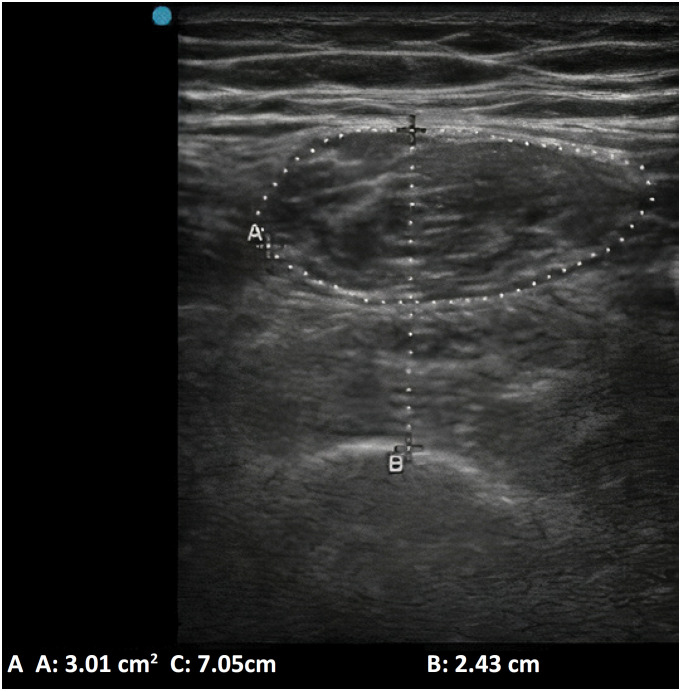
Ultrasound image acquisition

Ultrasonographic measurements of the cross-sectional area of the right rectus femoris (CSA) and the right quadriceps thickness (TQ) were performed by the first author, who was previously trained in these procedures. The training conducted for ultrasound assessment lasted approximately 4 hours and included theoretical and practical components. It was administered by an examiner with over 4 years of experience in ultrasound. A Sonosite Edge II portable ultrasound in B mode was used for the measurements, with frequencies adjusted between 5-10 MHz. A linear transducer in the transverse plane was positioned at two-thirds of the anterior superior iliac spine and the upper edge of the patella. Patient images were captured in the supine position with the right lower limb neutrally positioned, ensuring minimal compression with excess gel.^(19)^ The protocol involved obtaining three images and averaging the results for both measurements. To measure the TQ, a line was drawn between the highest point of the femur and superficial fascia, encompassing the rectus femoris and vastus intermedis muscles. During the CSA examination, the contour of the rectus femoris was delineated, excluding the fascia from the evaluation (Figure 1).^(20)^ These measurements were taken upon patient inclusion (D1) and five days later (D5), as well as at ICU discharge.

A) Area of the rectus femoris; B) Thickness of the rectus femoris and vastus intermedius.

The study's primary outcome was functional status at hospital discharge, which was evaluated via the BI. This index can be used to measure performance in various domains, such as feeding, grooming, bathing, dressing, bowel and bladder control, toilet use, transfers, ambulation, and stair climbing. The total scores range from 0 to 100, with higher scores indicating better functional status. Patients with a BI < 60^(21)^ were considered dependent. Two trained evaluators blinded to the ultrasound measurements determined the MRC score in the ICU and the BI score at hospital discharge.

The sample size calculation was conducted via the WinPEPI program (Programs for Epidemiologists for Windows) version 11.43 and was based on a study by Palakshappa et al.,^(22)^ which considered a significance level of 5%, a power of 80%, and a minimum correlation coefficient estimated at 0.40 between the change in the CSA of the rectus femoris from hospitalization to discharge from the ICU and functional status. Additionally, a 20% allowance was made for potential data loss. Thus, the minimum required sample size was 59 patients admitted to the ICU.

The Shapiro–Wilk test was used to assess the normality of continuous variables. Continuous variables are presented as the means ± standard deviations (SDs) or medians and interquartile ranges (IQRs). Categorical variables are presented as absolute numbers and percentages. Student's t-test or the Mann–Whitney test was applied to continuous variables, and the chi-square test was applied to categorical variables. The variation in the ultrasound data as a function of time was determined via a mixed linear model. The correlation between the ultrasound variables and functional status was assessed via the Pearson or Spearman correlation coefficient test. A multiple linear regression model was constructed to determine the impact of independent variables on the patient's functional status at hospital discharge. A priori variables were chosen based on their clinical plausibility concerning the outcome. These variables included age, sex, MRC score at awakening, SAPS 3 score, ICU length of stay, BI before ICU admission, corticosteroid usage, neuromuscular blocker usage, orthostatic capacity at ICU discharge, and TQ and CSA at ICU discharge. Stepwise forward regression was used to minimize overfitting. Multicollinearity was assessed via the variance inflation factor. The level of significance was set at p < 0.05. Statistical analyses were performed via the commercially available Statistical Package for Social Sciences (SPSS, Chicago, IL, USA) version 22.0.

## RESULTS

A total of 78 patients were initially enrolled, with 43 patients excluded due to mortality before hospital discharge, leaving 35 patients for the final analysis. The demographic and clinical data are detailed in table 1. This cohort comprised critically ill patients with an extended ICU stay (14.0; 9.0-26.0 days), among whom 13 (37.1%) were diagnosed with COVID-19. The ultrasonographic measurements of the CSA and TQ according to the functional status at hospital discharge are shown in table 2.

**Table 1:** Demographic and clinical data

		Independent (n = 15)	Dependent (n = 20)	p value
Age		55.3 ± 14.7	61.8 ± 15.7	0.225
Sex, male		10 (66.7)	11 (55.0)	0.728
BI before admission		100.0 (100.0 - 100.0)	100.0 (90.0 - 100.0)	0.043
Reason for admission				0.310
	Respiratory	2 (13.3)	1 (5.0)	
	Cardiovascular	4 (26.7)	3 (15.0)	
	Neurological	0	3 (15.0)	
	Sepsis	2 (13.3)	6 (30.0)	
	COVID-19	7 (46.7)	6 (30.0)	
	Other	0	1 (5.0)	
SOFA		10.3 ± 2.4	10.8 ± 3.4	0.642
SAPS 3		54.5 ± 22.8	60.4 ± 21.3	0.438
ICU-AW		9 (53.3)	20 (100)	0.001
Therapeutic support				
	Vasopressor	15 (100.0)	20 (100.0)	1.000
	Dialysis	7 (46.7)	8 (40.0)	0.741
	Corticoid	12 (80.0)	16 (80.0)	1.000
	NMBA	12 (80.0)	13 (65.0)	0.458
MV duration (days)		8.0 (5.0 - 14.0)	10.0 (4.3 - 22.0)	0.300
ICU LOS (days)		11.0 (7.0 - 20.0)	14.0 (10.0 - 29.8)	0.473
Hospital LOS (days)		28.0 (11.0 - 38.0)	37.5 (23.0 - 55.8)	0.092
BI at hospital discharge		75.0 (65.0 - 85.0)	20.0 (5.0 - 37.5)	< 0.001

BI - Barthel index; SAPS 3 - Simplified Acute Physiology Score 3; SOFA - Sequential Organ Failure Assessment; ICU-AW - intensive care unit-acquired weakness; NMBA - neuromuscular blocking agent; MV - mechanical ventilation; ICU - intensive care unit; LOS - length of stay. The results are expressed as the means ± standard deviations, medians (interquartile ranges), or n (%).

**Table 2:** Ultrasonographic measurements of the cross-sectional area of the right rectus femoris and the thickness of the right quadriceps according to functional status at hospital discharge

	Independent		Dependent	
	CSA	TQ	CSA	TQ
D1	3.3 ± 1.3	2.3 ± 0.6	2.6 ± 1.4	1.9 ± 0.8
D5	2.8 ± 0.9	2.5 ± 0.8	2.1 ± 0.7*	1.8 ± 0.4*
ICU discharge	2.2 ± 0.7	1.7 ± 0.5	1.7 ± 0.7*	1.4 ± 0.4*

CSA - cross-sectional area of the right rectus femoris; TQ - thickness of the right quadriceps; D1 - Day 1; D5 - Day 5; ICU - intensive care unit.

* p < 0.05 for comparisons between independent and dependent patients.

**Figure 2:**
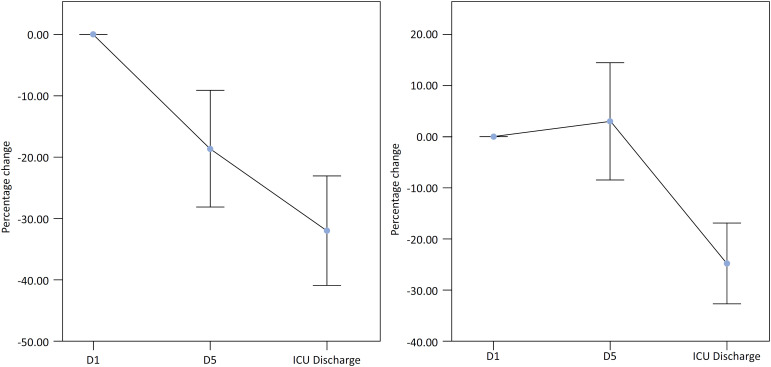
Changes in the cross-sectional area of the rectus femoris and thickness of the quadriceps during the intensive care unit stay.

On Day 1 (D1), the CSA measured was 2.9 ± 1.4cm^2^, showing a significant mean reduction of 18.6 ± 25.2%; p < 0.001 by Day 5 (D5) (Figure 2). From D5 to discharge from the ICU, an additional mean reduction of 15.3 ± 22.1%; p = 0.001, was subsequently observed. The TQ on D1 was 2.1 ± 0.8cm, with a mean variation of 3.0 ± 30.4%; p = 0.620 on D5. Notably, from D5 to ICU discharge, there was a mean reduction of 24.2 ± 20.3%; p < 0.001.

Percent variation in the transverse sectional area of the rectus femoris; B) percentage variation in the thickness of the quadriceps.

D1 - Day 1; D5 - Day 5; ICU - intensive care unit.

**Figure 3:**
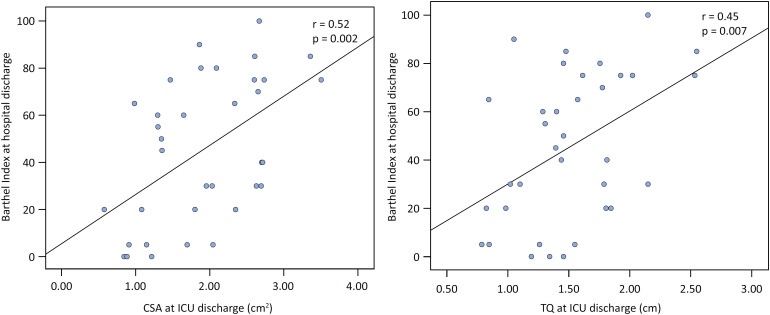
Correlations between the Barthel index at hospital discharge and A) the cross-sectional area of the right rectus femoris and B) the thickness of the right quadriceps at intensive care unit discharge.

At hospital discharge, the mean BI was 45.43 ± 30.78 points, with 20 (57.1%) patients categorized as dependent. Bivariate analysis revealed a moderate correlation between the primary outcome and CSA (r = 0.52; p = 0.002) as well as TQ (r = 0.45; p = 0.007) at ICU discharge (Figure 3). However, there was no correlation between BI at hospital discharge and the difference in CSA between D5 and D1 (p = 0.228) or the difference in TQ between D5 and D1 (p = 0.774). Multiple linear regression analysis demonstrated an independent association between the CSA at ICU discharge and the BI (Table 3).

CSA - cross-sectional area of the right rectus femoris; ICU - intensive care unit; TQ - thickness of the right quadriceps.

**Table 3:** Results of multiple linear regression for functional status assessed by the Barthel index

R^2^ = 0.694	Coefficient B	Standard error	Coefficient B adjusted	95%CI	p value
MRC on awakening	1.385	0.215	0.658	0.946 to 1.824	<0.001
Age	−0.587	0.220	−0.294	−1.035 to −0.138	0.012
CSA at ICU discharge	13.352	4.459	0.329	4.258 to 22.445	0.005

Other variables included in the model were SAPS 3 score, intensive care unit length of stay, sex, the Barthel index before intensive care unit admission, corticosteroid use, neuromuscular blocking agents, orthostatic capacity at intensive care unit discharge, and thickness of the right quadriceps at intensive care unit discharge.

95%CI - 95% confidence interval; MRC - Medical Research Council; CSA - cross-sectional area of the right rectus femoris; ICU - intensive care unit.

## DISCUSSION

We observed that ultrasound measurements of CSA at ICU discharge were significantly associated with functional status at hospital discharge, even after adjusting for several established risk factors influencing this outcome. In contrast, the variation in muscle mass during the initial five days of the ICU stay did not exhibit any association with functional status at hospital discharge.

Previous studies have demonstrated a correlation between ultrasound-measured muscle mass and functional capacity at various time points, such as 7 days,^(22)^ ICU discharge,^(20,23)^ and hospital discharge.^(14)^ Our study reinforces this correlation within a predominantly chronically critically ill patient population, where more than one-third had COVID-19, distinguishing it from prior research. Importantly, however, the significant correlation between the CSA measurements and functional status was moderate. The complexity of the relationship between muscle mass and function makes isolated muscle mass measurement unlikely to be a strong predictor of muscle function in critically ill patients.^(22)^

Patients identified as having an increased risk of functional disability at ICU discharge, especially through a reliable noninvasive tool,^(19)^ could benefit from more intense physical rehabilitation, potentially enhancing functional outcomes upon hospital discharge. However, a study that evaluated intense post-ICU physical rehabilitation without risk stratification revealed no difference in functional capacity or quality of life outcomes.^(24)^ This difference might be attributed to variations in intervention duration and the diverse nature of the patient population. Effectively identifying ICU-discharged patients at an elevated risk of functional disability could serve as an important factor in tailoring the intensity of subsequent physical rehabilitation.

The absence of a correlation between early ICU muscle mass loss in the ICU and functional status at hospital discharge in our study may be attributed to the prolonged duration between ICU admission and hospital discharge. Our patients had a median hospital stay of 30.0 (21.0 - 48.0) days. In contrast, Mayer et al. reported a relationship between CSA reduction from the first to the seventh day in the ICU and subsequent functional capacity at hospital discharge, with a median hospital stay of 11.2 (8.0–19.0) days.^(14)^ This implies that assessing muscle mass at ICU discharge is important for populations enduring extended ICU and hospital stays.

We assessed the muscle mass loss rate in critically ill patients using CSA as approximately 3.7% per day for the first 5 days, which was higher than the range of 2 - 3.3% for the first 7 - 10 days reported in other studies.^(14,20,22,23,25)^ This difference might be due to differing population characteristics. Our study included patients who had undergone MV for at least 48 hours, most of whom had received corticosteroids and neuromuscular blockers. We did not find a significant variation in the quadriceps thickness during the first five days of the ICU stay. A recent study revealed that the greatest variation in the thickness of the quadriceps muscle occurred after the sixth day of ICU stay, suggesting that the highest rate of muscle loss occurs during the resolution phase after metabolic stress exacerbates. ^(26)^ The area and thickness reductions in our study evaluated over the entire ICU stay were 28.9% ± 26.3% and 21.4% ± 24.2%, respectively. Even considering methodological and population differences, these results demonstrate significant muscle deterioration in critically ill patients.

Our study has several limitations. The study was conducted in a single center on a sample of predominantly chronic patients, including those with COVID-19, which may limit the generalizability of the results. Additionally, the limited sample size hindered the evaluation of ultrasonographic muscle mass variables' discriminatory power, preventing the establishment of a definitive cutoff point. Moreover, we did not utilize a gold standard measure, such as the six-minute walk test, to evaluate functional capacity. Instead, we opted for the BI as it provided functional status information and ease of application. Furthermore, although the reasons for admission to the ICU were not significantly different, likely due to the small number of patients included, there was a greater proportion of patients admitted for neurological reasons in the group of dependent patients. This disparity may have influenced the dependence assessment and the loss of muscle function. Finally, as the data were unsuitable for subgroup analysis, we analyzed only descriptive and correlational data.

## CONCLUSION

We observed that ultrasound measurements of the cross-sectional area at intensive care unit discharge were associated with functional status at hospital discharge. This finding underscores the potential utility of ultrasound in identifying patients at risk of functional loss, thereby enabling targeted interventions and potentially improving patient outcomes. However, further studies are warranted to rigorously evaluate this hypothesis.

## Data Availability

The datasets used and/or analyzed during the current study are available from the corresponding author upon reasonable request.
